# Comprehensive Monitoring and Benefit Evaluation of Converting Farmlands into Forests and Grasslands in China

**DOI:** 10.3390/ijerph19116942

**Published:** 2022-06-06

**Authors:** Shidong Li, Bing Wang, Sheng Zhang, Yingfa Chen, Guangshuai Zhao

**Affiliations:** 1 Management Office of Conversion of Farmlands to Forests and Grasslands, National Forestry and Grassland Administration, Beijing 100714, China; eastworld@sohu.com (S.L.); chenyingfa1@sina.com (Y.C.); 2 Institute of Forest Environmental Protection, Chinese Academy of Forestry, Beijing 100091, China; wangbing@caf.ac.cn; 3 Development Research Center, National Forestry and Grassland Administration, Beijing 100714, China; zhsh3360@sina.com

**Keywords:** conversion of farmlands to forests and grasslands, comprehensive benefits, monitoring and evaluation, indicator system

## Abstract

Conversion of farmlands to forests and grasslands (CFFG) is one of the major ecological projects with the largest investment, strongest policy, widest coverage and highest degree of participation in China, and even in the world. In order to scientifically evaluate the benefits and dynamic changes, better serve the decision-making, consolidate the achievements and promote the high-quality development of this project, it is of great significance to organize the monitoring and evaluation of its benefits. On the basis of reviewing and summarizing the monitoring and evaluation history of the benefits, this study established an indicator system for comprehensive monitoring and evaluation, composed of three components of benefits, 10 categories and 48 indicators, including 23 indicators of ecological benefits, 11 indicators of economic benefits and 14 indicators of social benefits. These methods of monitoring and evaluation are applied to the systematic and full coverage monitoring and evaluation of the national project of CFFG for the first time. There are four aspects of the innovation of this research: First, it is the first time that a comprehensive ecological, economic and social benefit evaluation indicator system has been established. Second, it is the first time that quantitative evaluation methods have been established. Third, it is the first comprehensive quantitative assessment of the CFFG project. Fourth, this is a full-scale evaluation of the project for the first time. The evaluation results show that the total value of the three benefits from the CFFG project is 2405.046 billion Yuan (354.4129 billion US$)·y^−1^, of which the ecological benefit is 1416.864 billion Yuan (208.7922 billion US$)·y^−1^, the economic benefit is 255.486 billion Yuan (37.649 billion US$)·y^−1^ and the social benefit is 732.696 billion Yuan (107.9717 billion US$)·y^−1^, accounting for 58.92%, 10.62% and 30.46%, respectively, of the total benefits. Our results provide detailed evaluation of the achievement and benefits of the CFFG project.

## 1. Introduction

Conversion of farmlands to forests and grasslands (CFFG) is an ecological project with the strongest policy, widest coverage and highest degree of participation in China, with efforts to protect and improve the environment to achieve sustainable development goals (SDGs) [1,2]. The first phase of this CFFG project was launched in 1999, and the second round continued in 2014. By the end of 2019, the total project area arrived at 34.33 million ha, including 13.73 million ha of converted forests and grasslands, 17.53 million ha of afforested land and 3.07 million ha of closed forests. The Chinese central government has invested RMB 517.4 billion Yuan (76.2452 billion US$) to this project, which covers 2435 counties in 25 provinces. The afforestation of this project accounts for 40.5% of the total afforestation area of key ecological projects in the country during the same period in China, and more than 4% of the global greening area. The forest cover in the project area has increased by more than 4% on average and directly benefited 41 million households and approximately 158 million farmers [3]. Therefore, monitoring and evaluating the ecological, economic and social benefits of the CFFG project is an important task of forest resource accounting, which can scientifically reflect the achievement of this ecological project.

There are many reports on forest resource accounting and benefit monitoring, and evaluation at home and abroad [4,5,6,7,8,9]. In 2004, 2013 and 2016, national forestry authorities have conducted national forest resource accounting three times, but the economic benefit has only evaluated the value of forest land and trees, and the social benefit only included one indicator of cultural value in the third accounting, which cannot reflect the realized achievement and benefits of this project [10,11,12]. The State Forestry Administration of China has also evaluated the benefit of the CFFG project five times from 2013 to 2017 [13,14,15,16,17]. In terms of the components of monitoring and assessment, the previous four times focused on ecological benefit assessments of the projects and, only in 2017, were ecological, economic and social benefit assessed for 14 contiguous poor areas of the project [14]. In terms of the methods of monitoring and assessment, the project assessment was mainly qualitative, lacking systematic and comprehensive quantitative assessments [14,18,19]. In terms of the scope, the monitoring and assessment were conducted in part of the project areas from 2013 to 2017 [13,14,15,16,17]. Therefore, the monitoring and assessment were based on an incomplete set of indicators, insufficient quantification and incomplete coverage. To address these issues, the objectives of this study are: (1) to establish a systematic and scientific monitoring and assessment index system for the comprehensive benefits; (2) to establish a scientific and feasible calculation method by its own characteristic; and (3) to comprehensively and quantitatively assess the benefits, and make an objective evaluation for the effectiveness assessment of major ecological projects and the contribution of ecological protection in China.

## 2. Materials and Methods

### 2.1. Methods

The evaluation index system of ecological, economic and social benefits of the project was established comprehensively through enriching the socio-economic indices, improving the evaluation indices of primary, secondary and tertiary industries, and selecting comprehensive indices for social benefits such as developing social undertakings, optimizing social structure, improving social service functions and promoting the development of social organizations [20,21]. This study adopted reasonable value calculation methods such as the market method (including simulated market), income method (including expected income), and cost method (including opportunity cost), and expands and innovates the theory, method, and index system of China’s forest resources accounting index system to ensure the authority and accuracy and credibility of the research results.

### 2.2. Data Source

In this study, we comprehensively consider data sources and statistical analysis in the process of establishing the index system and assessment methods. Only reliable and accurate data were used, and the statistical analysis was standardized and programmed.

(1) Ecological benefit data sources and statistical analysis

The ecological benefit assessment of the CFFG project is divided into two parts: physical assessment and value assessment. The physical assessment is based on a forest resource continuous inventory dataset (2435 samples) and long-term forest ecological observation dataset (407 observation stations) of the CFFG project. The value assessment requires a social public dataset, in addition to the above two datasets. The above three data sources are applied to a series of assessment formulas to obtain the ecological benefit assessment results of the project, which is the coupled integration of the above data sources.

(2) Social and economic benefits data sources and statistical analysis

This study is the first time that a full-coverage questionnaire and a quantitative assessment (2435 samples) has been adopted, covering 287 municipalities (including prefecture-level units) and 2435 counties (including county-level units) in 25 provinces (or autonomous regions and municipalities) and Xinjiang Production and Construction Corps across China. We conducted questionnaire investigation at the county-level, summarized at the provincial-level, and, finally, undertook statistical analyses. Based on the index system for monitoring and evaluating the social and economic benefits of the project, an operational, fillable and accurate social and economic benefit evaluation questionnaire was designed, and the total social and economic values were the sum of all social and economic indices of all the counties of the project, respectively.

## 3. Results

### 3.1. Monitoring and Evaluation Indicator System for the CFFG Project

#### 3.1.1. Ecological Benefit Monitoring and Evaluation Index System

**Figure 1:**
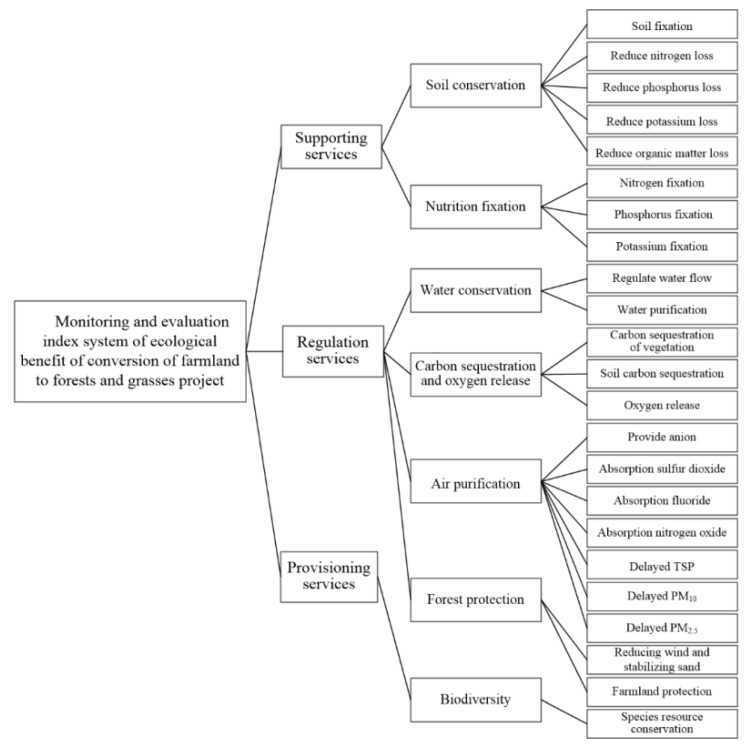
Monitoring and evaluation index system of ecological benefit of the CFFG project.

According to national forest resource assessment standards include Observation and Methodology for Long-term Forest Ecosystem Research (GB/T 33027-2016) [22], Indicators System for Long-term Observation of Forest Ecosystems (GB/T 35377-2017) [23], Specification for the Assessment of Forest Ecosystem Service (GB/T 38582-2020) [24], Specification for Construction on Long-term Observation Research Station of Forest Ecosystems (GB/T40053-2021) [25], and the specifications of monitoring and evaluation of the CFFG project such as Evaluation in Project for the Construction of Conversion of Farmlands to Forests (GB/T23233-2009) [26], Specification of Monitoring and Evaluation of Ecological Benefits of Converting Farmlands to Forests Project (LY/T2573-2016) [27]. The ecological benefit monitoring and assessment index system includes three categories and seven aspects, i.e., supporting services (soil conservation and nutrient fixation), regulation services (water conservation, carbon sequestration and oxygen release, air purification and forest protection) and provisioning services (biodiversity), covering 23 indicators [5,28] (Figure 1).

#### 3.1.2. Economic Benefit Monitoring and the Evaluation Index System

**Figure 2:**
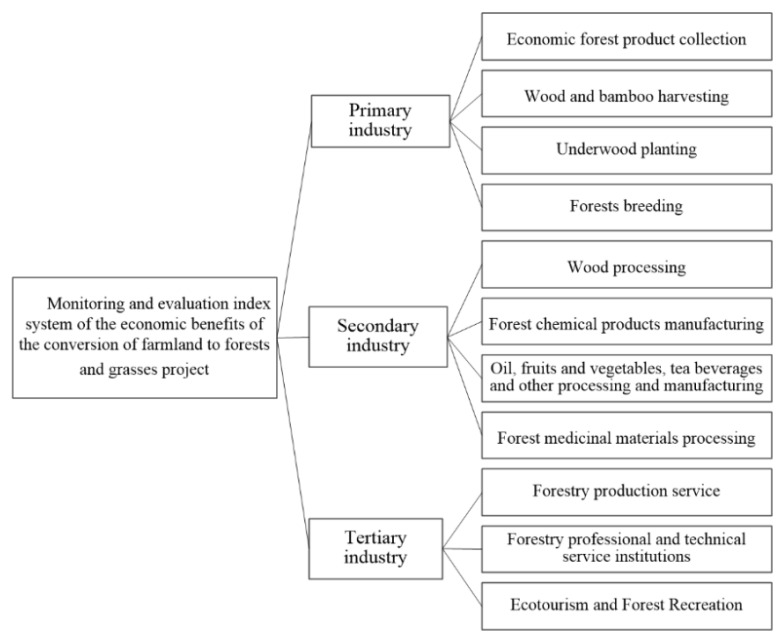
Monitoring and evaluation index system of the economic benefits of the CFFG project.

CFFG is not only an ecological project, but also a project for poverty alleviation, rural industrial structure adjustment, and promotion of rural revitalization, which is a vivid practice of the concept of “lucid waters and lush mountains are invaluable assets”. According to the relevant standards include Evaluation in Project for the Construction of Conversion of Farmlands to Forests (GB/T23233-2009) [26], Technical Manual on Assessing Forest Resources Assets (LY/T 2407-2015) [29], Indicators for Monitoring and Assessment of Socio-economic Impacts of the Program for Conversion of Farmlands to Forests (LY/T1757-2008) [30], and with reference to the nearly 20 years of research foundation and monitoring practice accumulated by the Monitoring and Assessment of Socio-economic Impacts of China’s Key Forestry Program launched in 2002, the monitoring indicator system selected for the economic benefits of the CFFG project includes 11 evaluation indicators in three categories: primary, secondary and tertiary industries [20,21] (Figure 2).

#### 3.1.3. Social Benefit Monitoring and Evaluation Index System

**Figure 3:**
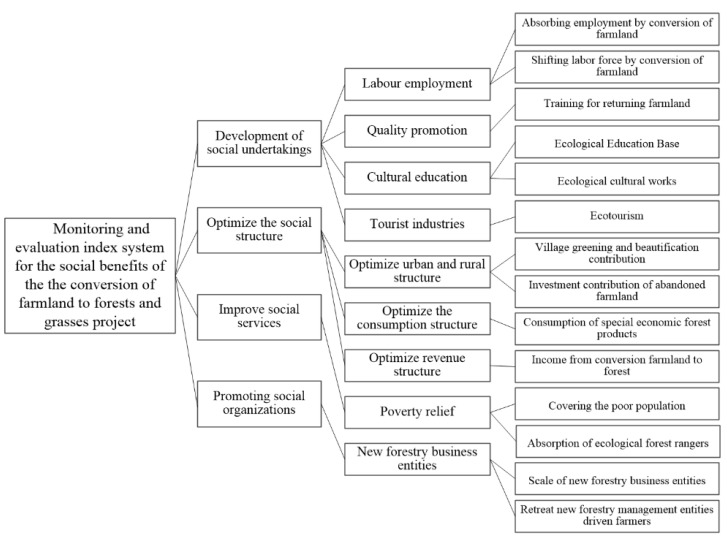
Monitoring and evaluation index system for the social benefits of the CFFG project.

The CFFG is also a far-reaching benevolent project and a social project that has received wide attention both at home and abroad. Based on Evaluation in Project for the Construction of Conversion of Farmlands to Forests (GB/T23233-2009) [26], Indicators for Monitoring and Assessment of Socio-economic Impacts of the Program for Conversion of Farmlands to Forests (LY/T1757-2008) [30], and with reference to the nearly 20 years of research foundation and monitoring practice accumulated by the Monitoring and Assessment of Socio-economic Impacts of China’s Key Forestry Program, the monitoring indicator system selected for social benefits includes 14 monitoring indices in four categories (development of social undertakings, optimize the social structure, improve social service and promoting social organizations) [20,21] (Figure 3).

### 3.2. Monitoring and Benefit Evaluation Methods of the CFFG Project

#### 3.2.1. Ecological Benefit Monitoring and Evaluation Methods

Since the monitoring and evaluation of ecological benefits of the CFFG was carried out in 2012, a comprehensive monitoring system has been formulated. The ecological benefit monitoring stations were mainly based on the Chinese Forest Ecological Research Network (CFERN), including 108 national ecological research stations, more than 230 auxiliary observation points and more than 8500 fixed sample plots, simultaneously absorbing the relevant data collected by 69 provincial special monitoring stations for conversion of farmlands to forests. After the ecological function monitoring and zoning of the national CFFG project, the main methods, including the ecological continual inventory system, coupled integration of multiple datasets based on the distributed measurement method and ecological functional coefficient correction assessment model were adopted to measure the amount of ecological benefit (Table 1) [10,12,31,32].

#### 3.2.2. Economic Benefit Monitoring and Evaluation Methods

In August 2002, the State Forestry Administration launched the project of “Monitoring the Socio-economic Benefits of CFFG Project”, which has continually monitored the socio-economic benefits of this project for 19 years. The scope of socio-economic assessment in this study was expanded from 100 sample counties to all counties of the CFFG project. The main method was to develop county-level questionnaires corresponding to 11 assessment indicators in three categories, with comprehensive surveys at the county level, weighted summaries at the provincial level, and then unified summaries for calculation (Table 2).

#### 3.2.3. Social Benefit Monitoring and Evaluation Methods

Based on 14 monitoring indicators of nine components in four categories of the social benefit monitoring and assessment, an operational, fillable and accurate economic benefit assessment questionnaire was designed. The survey form filled out at the county level and the summary survey form carried out at the province level were used in this monitoring and assessment. The statistics were then analyzed and the evaluation was measured. The computation methods were provided in Table 3.

### 3.3. Comprehensive Benefits of the CFFG Project

**Figure 4:**
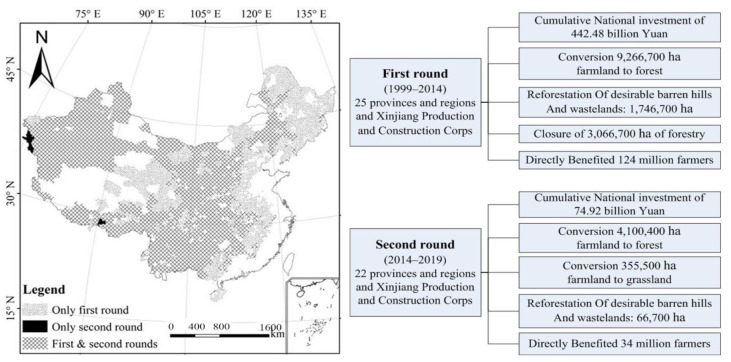
Spatial patterns and achievements in two phases of the CFFG project.

The CFFG project has become a major ecological undertaking with the most capital investment, the largest construction scale, the strongest policy and the highest degree of participation in China and over the world, and it has also achieved great comprehensive benefits (Figure 4). In order to quantify and facilitate comparative analysis of the comprehensive benefits of the project, all three major benefits of this study were measured quantitatively in terms of economic value. The study shows that the total value of ecological, economic and social benefits is 2405.046 billion Yuan (354.4129 billion US$)·y^−1^ in the 25 project provinces (regions) and Xinjiang Production and Construction Corps in 2019.

#### 3.3.1. Results of Ecological Benefits

The amounts of the ecological benefits in terms of quantity from the CFFG project were: water connotation 44.005 billion m^3^·y^−1^, carbon sequestration (oxygen release) 55.7026 (132.6682) million tons·y^−1^, purification of the atmospheric environment 540.1909 million tons·y^−1^, wind control and sand fixation 837.25 million tons·y^−1^, soil fixation 709.4355 million tons·y^−1^, fertilizer retention 28.907 million tons·y^−1^, and forest nutrient fixation 1.2134 million tons·y^−1^.

The total value of ecological benefits is 1416.864 billion Yuan (208.7922 billion US$), of which the largest value was water conservation at 463,022 million Yuan (68,231 million US$)·y^−1^, accounting for 32.68% of the ecological value; the second was purifying the atmosphere at 310,175 million Yuan (45,708 million US$)·y^−1^, accounting for 21.89% of the ecological value; the third was carbon sequestration and oxygen release at 223,017 million Yuan (32,864 million US$)·y^−1^, accounting for 15.74% of the ecological value (Table 4).

#### 3.3.2. Results of Economic Benefits

The total value of economic benefits was 255.486 billion Yuan (37.649 billion US$) from the CFFG project, of which the values of primary industry, secondary industry and tertiary industry were 148,305, 65,453 and 41,728 million Yuan (21,855, 9645 and 6149 million US$), respectively, accounting for 58.05%, 25.62% and 16.33%, in turn (Table 5).

#### 3.3.3. Results of Social Benefits

The total value of social benefits was 732.696 billion Yuan (107.9717billion US$) in 2019 from the CFFG project, of which the values of developing social undertakings, optimizing social structure, improving social service functions and promoting the development of social organizations accounting for 61.06%, 33.84%, 0.85% and 4.24%, respectively, of the total social benefit value (Table 6).

## 4. Discussion

The comprehensive benefit of the CFFG project in 2019 was 2405.046 billion Yuan (354.4129 billion US$)·y^−1^, which was 4.65 times of the accumulated investment of the central government (517.4 billion Yuan, 76.2452 billion US$), fully reflecting the huge benefits of this project. The largest benefit of the project was in terms of the ecological benefit, accounting for 58.92% of the total, especially for the top four functions, i.e., water conservation, purification of atmospheric environment, carbon sequestration and oxygen release and biodiversity, accounting for 84.91% of the total ecological benefits, fully reflecting the ecological benefits of the CFFG project as the “green reservoir”, “oxygen bar reservoir” and “carbon reservoir”, as well as the biological gene pool [45,46,47,48]. These aspects can achieve improvement of the ecological environment and biodiversity conservation, and are an important part of the management and ecological restoration of mountains, water, forests, fields, lakes and grasslands [1], It was also the fulfillment of multiple sustainable development goals (SDGs), including targets for poverty reduction, good health and well-being, clean water and sanitation, sustainable cities and communities, climate action, and terrestrial biodiversity conservation [2,49]. Social benefits were the second most important benefits, accounting for 30.46% of the comprehensive benefits. The CFFG project not only effectively improved the ecological environment, but also released the rural labor force, promoted labor force transfer and employment structure adjustment [50,51], while absorbing the rural population into nearby employment, promoted the greening and beautification of the countryside, and provided the foundation and guarantee for green health [52,53]. In addition, it can also optimize the allocation of factors, which advances the change in agricultural planting structure, and thus affects the adjustment of the whole industrial structure and improves the living standard of farmers, etc. [54,55]. All these are important elements to achieve rural revitalization.

From different regions, the values of ecological benefits of water conservation, purification of the atmospheric environment, carbon sequestration and oxygen release of the CFFG project were substantially higher in the middle and upper reaches of Yangtze River and Yellow River than in other regions, due to abundant precipitation or large project area in these regions [56,57]. The higher the precipitation in the area of the project, the more the water storage capacity in the soil and the stronger the dust retention function, while the quantities of water conservation, carbon sequestration and dust retention in the larger project area were also higher. The social benefit values of the top three provinces were Sichuan, Hubei Provinces and Chongqing Municipality, accounting for 32.77% of the total social benefit value. The development of the ecotourism industry delivered by the implementation of the CFFG project has contributed to the increase in the value of social benefits. In addition, the total social benefits of the project were proportionate to the size of the ecotourism development. In addition, the change in labor employment structure caused by the project implementation has also influenced the social benefits to a large extent [58,59]. The top three provinces, in terms of the values of economic benefits formed by the project, were Guangxi, Chongqing and Sichuan, accounting for 34.58% of the total economic benefit value. The development of fallowed economic forests and the understory forest economy was the main reason for the rise in the economic benefits of forest industry [60]. In addition, the economic benefits of the CFFG project in different areas were consistent with the performance of economic forest harvesting and the size of the understory forest economy. In addition, the development of the timber industry largely affected of the economic benefits of the CFFG project.

Compared with other ecological projects, the CFFG project had relatively higher ecological benefits. The annual average ecological benefit of the CFFG project was 41,300 Yuan (6086 US$)·ha^−^^1^, accounting for ca. 84% of the national average (49,000 Yuan (7221 US$)·ha^−^^1^·y^−1^) [10], and 4.54 times the average benefit of the Three North Shelterbelt Project in Phase V (9100 Yuan (1341 US$)·ha^−^^1^·y^−1^) [61], and 2.33 times the benefit of the Key State-owned Forest Areas in Northeast and Inner Mongolia(17,700 Yuan (2608 US$)·ha^−^^1^·y^−1^) [62], indicating that the implementation of the CFFG project in sloping lands with poor stand conditions was effective. In particular, in terms of carbon sequestration and oxygen release, most of the plantations of the CFFG accumulated rapid forest stock [63], and sequestrated carbon and released oxygen in more than 18% of the total forest ecosystems in the country in the same period [10], accounting for 5.86 times as much as the Three North Shelterbelt Project in Phase V [61] and 2.41 times as much as the Key Sate-owned Forest Areas in Northeast and Inner Mongolia [62]. The carbon sequestration of the CFFG project could offset about 2.18% of the national CO_2_ emissions in 2018 [64], which is important for China to adapt to climate change and achieve the goal of “carbon neutrality”. Of course, in addition to the differences brought by the construction of the project itself, there were also important influences of factors such as the location of the project, natural environment and resource background.

Although the comprehensive monitoring and benefit evaluation of the CFFG project has achieved phased results, there are still some problems. First, the number, distribution and monitoring quality of ecological stations for obtaining ecological data need to be further optimized and improved. Second, the content of monitoring and evaluation should be further detailed based on project examples, including quantifying the ecological, social and economic benefits of different tree species, technologies and management models, so that the evaluation results will be more targeted to guide specific practices of the CFFG project. Third, ecological and socio-economic monitoring are still two independent parts. How to better carry out the coupling research between the two is very important for the future. In addition, forest resource accounting attaches more importance of the public benefit assessment, which was different from the market exchange value. Therefore, its theories and methods still need to keep up with the times. Among the three major ecological, economic and social values of the CFFG project, ecological and social benefits were public services, such as water conservation, atmospheric purification, carbon dioxide fixation and oxygen release, etc. After converting them into economic values according to the amount of human labor required, the monetary values were generally larger, but they were not economic benefits in the traditional sense. Although public services may be assessed by the methods of a simulated market, alternative market, and opportunity cost, and by the monetary exchange value, the simulated market, alternative market, and opportunity cost methods for the same public services can also be different, and the assessment results vary greatly. As a result, the value of public services was still different from the real economic return, and thus was only a reference for investment cost analysis. Meanwhile, forest resource accounting was also a process of keeping up with the times, such as the value assessment of carbon sinks, and there was currently an artificial carbon sequestration cost method, carbon tax method, carbon sink trading method (the market is currently under construction), and afforestation cost method, as well. This study adopted the artificial carbon sequestration cost method, but several years later, when the national carbon sink market is mature, it may be more reasonable to adopt the carbon sink trading method.

## 5. Conclusions

This study showed that the comprehensive value of the CFFG project was 2405.046 billion Yuan (354.4129 billion US$)·y^−1^ in 2019, of which the ecological, economic and social benefits were 1416.864 billion Yuan (208.7922 billion US$)·y^−1^, 255.486 billion Yuan (37.649 billion US$)·y^−1^ and 732.696 billion Yuan (107.9717 billion US$)·y^−1^, accounting for 58.92%, 10.62% and 30.46% of the comprehensive benefits, respectively. The sum of the ecological and social benefit values, accounting for 89.38% of the total assessed value, coincide with the national overall objectives of the ecological restoration project, and are fully consistent with the aims of other ecological projects, virtuous projects and popularity-win projects. 

The evaluation indicator systems and methods for the ecological, economic and social benefits are constructed based on 10 years and 20 years of monitoring practice, respectively, which are proved to be in line with the project practice and will provide reference for global ecological governance evaluation. This research is the first time that the economic value of ecological benefits, economic benefits and social benefits have been comprehensively and quantitatively evaluated, based on large samples (2435 sample counties and 407 observation stations). It is an important part of the CFFG project, and it also makes an important contribution to the green development of the world. Meanwhile, the results of this research will be directly used to support the Chinese government in scientifically formulating reasonable ecological subsidy standards, in the high-quality development of a master plan for the CFFG, and in revising relevant policies and standards, for example, Regulations on Converting Farmlands to Forests, Evaluation in Project for the Construction of Conversion of Farmlands to Forests (GB/T23233-2009), Indicators for Monitoring and Assessment of Socio-economic Impacts of the Program for Conversion of Farmlands to Forests (LY/T1757-2008), etc.

## Figures and Tables

**Table 1:** Evaluation method for the ecological benefit value of the CFFG project.

Service Categories	Function Categories	Indicator Categories	Calculation Formula and Parameter Description
Supportive services	Soil conservation	Soil fixation	$U_{sf}=C_{s}\cdot G_{sf}/\rho$ where *U_sf_* is the annual soil fixation value of the assessed stand (Yuan·y^−1^); *C_s_* is the cost required to dig and transport a unit volume of soil (Yuan·m^−3^); *G_sf_* is the annual soil fixation amount (tons·y^−1^); and *ρ* is soil bulk density (g·cm^−3^)
		Reducing nitrogen loss	$U_{of}=C_{N}C_{1}/R_{1}+G_{P}C_{1}/R_{2}+G_{K}C_{2}/R_{3}+G_{of}C_{3}$ where *U_of_* is the annual reducing nutrient loss value of the assessed stand (Yuan·y^−1^); *G_N_*, *G*_*P*_, *G_K_* and *G_of_* are the reduction in nitrogen, phosphorus potassium and soil organic matter loss (tons·y^−1^) respectively, due to soil fixation by the assessed stand; *C*_1_, *C*_2_ and *C*_3_ are the fertilizer price of diammonium phosphate, potassium chloride and organic matter (Yuan·ton^−1^) respectively; *R*_1_, *R*_2_ and *R*_3_ are the nitrogen content of diammonium phosphate fertilizer (%), phosphorus content of diammonium phosphate fertilizer (%) and potassium content of potassium chloride fertilizer (%), respectively.
		Reducing phosphorus loss	
		Reducing potassium loss	
		Reducing organic matter loss	
	Nutrient fixation	Nitrogen retention	$U_{N}=G_{N}C_{1}/R_{1}$ where *U*_*N*_ is the value of nitrogen retention in the assessment year (Yuan·y^−1^); *G*_*N*_ is the annual nitrogen retention in the assessment stand (tons·y^−1^); *C*_1_ is the price of diammonium phosphate fertilizer (Yuan·ton^−1^); *R*_1_ is the nitrogen content of diammonium phosphate fertilizer (%);
		Phosphorus retention	$U_{P}=G_{P}C_{1}/R_{2}$ where *U*_*P*_ is the value of phosphorus retention in the assessment year (Yuan·y^−1^); *G*_*P*_ is the amount of phosphorus retention in the assessment forest year (tons·y^−1^); *C*_1_ is the price of diammonium phosphate fertilizer (Yuan·ton^−1^); *R*_2_ is the phosphorus content of diammonium phosphate fertilizer (%).
		Potassium retention	$U_{K}=G_{K}C_{2}/R_{3}$ where *U_K_* is the value of potassium retention in the assessment year (Yuan·y^−1^); *G_K_* is the amount of potassium retention in the assessment year (tons·y^−1^); *C*_2_ is the price of potassium chloride fertilizer (Yuan·ton^−1^); and *R*_3_ is the potassium content of potassium chloride fertilizer (%);
Regulative services	Water Conservation	Regulation of water volume	$U_{A}=G_{A}\cdot C_{V}$ where *U_A_* is the value of the annual regulation of water volume in the assessed forest (Yuan·y^−1^); *G_A_* is the annual regulation of water volume (m^3^·y^−1^); *C_V_* is the reservoir capacity construction cost (Yuan·m^−3^)
		Water purification	$U_{PU}=G_{PU}\cdot K_{W}$ where *U_PU_* is the annual value of water purified by the assessed forest (Yuan·y^−1^); *G_PU_* is the annual volume of water regulation in the assessed forest stand (m^3^·y^−1^); *K_W_* is the cost of water purification (Yuan·m^−3^)
	Carbon sequestration and Oxygen release	Carbon sequestration	$U_{C}=G_{C}\cdot C_{C}$ where *U_C_* is the annual value of carbon sequestration in the assessed stand (Yuan·y^−1^); *G_C_* is the potential annual carbon sequestration of the assessed stand ecosystem (tons·y^−1^); and *C_C_* is the price of carbon sequestration (Yuan·ton^−1^)
		Oxygen release	$U_{O}=G_{O}\cdot C_{O}$ where *U_O_* is the value of annual oxygen release from the assessed stand (Yuan·y^−1^); *G_O_* is the annual oxygen release from the assessed stand (tons·y^−1^); and *C_O_* is the price of manufacturing oxygen (Yuan·ton^−1^).
	Purifying the atmosphere	Providing negative ions	$U_{NI}=5.256\times 10^{15}A\cdot H\cdot K_{NI}\cdot (Q_{NI}-600)\cdot F/L$ where *U_NI_* is the annual value of negative ions provided by the assessed stand (Yuan·y^−1^); *K_NI_* is the cost of negative ion production (Yuan·each^−1^); *Q_NI_* is the concentration of negative ions in the assessed stand (pcs·cm^−3^); *L* is the life span of negative ions (min); *H* is the stand height (m); *A* is the stand area (ha); *F* is the forest ecological function correction factor
		Sulfur dioxide absorption	$U_{SD}=G_{SD}\cdot K_{SD}$ where *U_SD_* is the annual value of SO_2_ absorbed by the assessed forest stand (Yuan·y^−1^); *G_SD_* is the annual amount of SO_2_ absorbed by the assessed forest stand (kg·y^−1^); *K_SD_* is the treatment cost of SO_2_ (Yuan·kg^−1^)
		Fluoride absorption	$U_{F}=G_{F}\cdot K_{F}$ where *U_F_* net is the annual water purification value of the assessed forest Yuan·y^−1^); *G_F_* is the annual fluoride uptake of the assessed forest stand (kg·y^−1^); *K_F_* is the treatment cost of fluoride (Yuan·kg^−1^)
		Nitrogen oxide absorption	$U_{NO}=G_{NO}\cdot K_{NO}$ where *U_NO_* is the annual value of water purified by the assessed forest (Yuan·y^−1^); *G_NO_* is the annual amount of NO_x_ absorbed by the assessed forest stand (kg·y^−1^); *K_NO_* is the treatment cost of NO_x_ (Yuan·kg^−1^)
		Reducing total suspended particulates (TSP)	$U_{L}=(G_{TSP}-G_{PM_{10}}-G_{PM2.5})\cdot K_{TSP}+U_{PM_{10}}+U_{PM2.5}$ where *U_L_* is the assessed stand annual potential reducing dust value (Yuan·y^−1^); *G_TSP_* is the assessed stand annual reduced TSP volume (Yuan·kg^−1^); *G_PM10_* is the assessed stand annual reduced PM_10_ volume (Yuan·kg^−1^); *G_PM2.5_* is the assessed stand annual reduced PM_2.5_ volume (Yuan·kg^−1^); *U_PM10_* is the assessed stand annual reduced PM_10_ value (Yuan·y^−1^). *U_PM2.5_* is the value of annual reduced PM_2.5_ in the assessed forest stand (Yuan·y^−1^); *K_TSP_* is the cost of dust reduction and cleanup (Yuan·y^−1^)
		Reducing PM_10_	$U_{PM10}=G_{PM10}\cdot C_{PM10}$ where *C_PM10_* is the cost of PM10 cleanup (Yuan·kg^−1^)
		Reducing PM_2.5_	$U_{PM2.5}=G_{PM2.5}\cdot C_{PM2.5}$ where *C_PM2.5_* is the cost of PM_2.5_ cleanup (Yuan·kg^−1^)
	Forest Protection	Wind and sand prevention	$U_{PW}=G_{PW}\cdot K_{PW}$ where *U_PW_* is the value of wind and sand control in the assessed stands (Yuan·y^−1^); *K_PW_* is the cost of sand fixation by straw-checkerboard (Yuan·ton^−1^); and *G_PW_* is the mass of sand fixation in the assessed forests (tons·y^−1^).
		Farmlands protection	$U_{FP}=V_{a}\cdot M_{a}\cdot K_{a}\cdot A_{F}$ where *U_FP_* is the value of the farmlands protection function of the assessed stand (Yuan·y^−1^); *V_a_* is the price of crops and pasture (Yuan·kg^−1^); *M_a_* is the average increase in crop and pasture production (kg·ha^−1^·y^−1^); *K_a_* is the conversion coefficient that average 1 ha of farmlands shelterbelt can protect 19 ha of farmlands; *A_F_* is the area of farmlands shelterbelt (ha)
Provisional services	Biodiversity	Species conservation	$U_{Total}=\left(1+0.1\sum_{m=1}^{x}E_{m}+0.1\sum_{n=1}^{y}B_{n}+0.1\sum_{r=1}^{z}O_{r}\right)\cdot S_{I}\cdot A\left(i=1,\;2,\;\cdots ,\;n\right)$ where *U_Toatl_* is the annual value of biodiversity conservation in the assessed stand (Yuan·y^−1^); *E_m_* is the endangerment index of species *m* in the assessed stand or region; *B_n_* is the endemic species index of species *n* in the assessed stand or region; *Or* is the old tree age index of species *r* in the assessed stand or region; *x* is the number of species for calculating the endangerment index; *y* is the number of species for calculating the endemic species index; *z* is the number of species for calculating the old tree age index; *S_l_* is the amount of species diversity conservation value per unit area (Yuan·ha^−1^·y^−1^); *A* is the area of the stand (ha)

**Note:** All constant parameters such as prices, coefficients and indices in the table are quoted from the National Report on Monitoring the Ecological Benefits of the CFFG Projects (2017) [14].

**Table 2:** Evaluation method of the economic benefit value of the CFFG project.

Indicators		Calculation Formula and Parameter Description
Primary Industries	Economic Forest Products	=Σ(the amount of harvesting fruits × price + amount of harvesting nuts and oilseeds × price + amount of harvesting tea and beverages ×price + amount of harvesting medicinal herbs × price)
	Timber and bamboo harvesting	=Σ(Wood harvesting volume × price + bamboo harvesting volume × price)
	Forest Plantation	=Σ(Production of forest mushrooms × price + production of food under forests × price + production of forest vegetables × price + production of forest tree seedlings × price + production of forest medicinal herbs × price)
	Forestry farming	=Σ(chicken raising under forests × price + forest duck raising under forests × price + goose raising under forests × unit price) + Σ(forest pig production × price + forest cattle production × price + forest sheep production × price) + Σ(forest bee and honey production × price)
Secondary Industries	Wood processing	=Σ(volume of wood processing × price) × contribution factor of fallow to wood processing
	Forestry chemical products manufacturing	=Σ(Volume of forest chemical product manufacturing × price) × contribution factor of fallowing to forest chemical product manufacturing
	Woody oilseeds, fruits and vegetables, tea beverages and others processing and manufacturing	=Σ(amount of oilseed processing and manufacturing × price) × contribution coefficient of fallow to woody oilseed processing and manufacturing + Σ(amount of fruits and vegetables processing and manufacturing × price) × contribution coefficient of fallow to fruits and vegetables processing and manufacturing + Σ(amount of tea and beverage processing and manufacturing ×price) × contribution coefficient of fallow to tea and beverage processing and manufacturing
	Forest medicines	=Σ(sum of herb medicines processed and manufactured × price) × contribution factor of fallow to herb medicines processing
Third industries	Forestry production services	=ΣBusiness income of forestry production service agencies × contribution factor of fallow to forestry production service agencies
	Forestry professional technical Services	=Σbusiness income of professional forestry technical service agencies × contribution coefficient of fallow to professional forestry technical service agencies
	Ecotourism and forestry recreation services	=Σbusiness income of ecotourism base × contribution coefficient of fallow to ecotourism + Σbusiness income of forest recreation base × contribution coefficient of fallow to forest recreation

**Note:** All the prices and the contribution coefficients of fallow to each industry were obtained from the survey forms submitted by different counties for monitoring the economic benefits of conversion of farmlands to forest and grassland projects, with reference to the research results of Chen et al. [33]. All the calculations are based on the county level.

**Table 3:** Evaluation method of social benefit value of the CFFG project.

Indicators			Calculation Formula and Parameter Description
Development of Social Business	Labor Employment	Employment in grain for grain project	=Σ(Average wage income of fallow farmers × number of people directly employed) × increase coefficient of employment in fallow farmers + Σnumber of people employed near the fallow farmlands × per capita transportation, accommodation and maintenance costs for working outside
		Transferring of labor from farming	=Σ(Number of laborers transferred × coefficient of contribution of fallow to labor transferring × average working wage level)
	Labor quality Enhancement	Employee Training	=Σ(Cumulative cost of farming skills training + Cumulative cost of listening to farming policy advocacy + Cumulative cost of farming employment training)
	Cultural Education	Ecological education base	=ΣNumber of people received employment education in display and education bases for fallowed farmers × cultural and educational input per capital
		Eco-cultural productions	=Σ(Number of cultural works displayed × pricing + number of cultural works performed × ticket price + number of cultural works published × price)
	Tourism Career	Ecotourism	=ΣEcotourism output value × contribution coefficient of fallow to ecotourism × forest tourism industry driving coefficient
Optimize the social structure	Optimizing urban and rural structure	Village greening and beautification contribution	=ΣNumber of people benefited by greening health × 0.3 × per capital medical cost
		Project investment contribution	=ΣTotal investment in CFFG project in × investment multiplier of the project
	Optimizing consumption structure	Special economic forest products consumption	=ΣNumber of people consuming special economic forest products × 0.13 × per capital medical expenses
	Optimizing revenue structure	Income from CFFG project	=ΣForestry income of households on fallowed farmlands
Improving social service functions	Poverty alleviation through fallow	Covering the poor	=ΣFallowed area of poor households× fallowed subsidy standard × fallowed project investment multiplication factor = Σ(fallowed area × average percentage of fallowed area of poor households) ×fallowed subsidy standard × fallowed project investment multiplication factor
		income from forest rangers	=Σ(ratio of the number of forest rangers in each province to the total area of fallow farmlands and forest land in each province) × financial subsidy standard for forest rangers
Promoting the development of social organizations	New Forestry Management entities	The size of the new forestry entity of the fallow project	=ΣNew forestry management entities in each province fallowed forestry output value × new forestry management entity driving coefficient
		Households benefited by fallowed new forestry business entities leading	=Σ(value of social stability + value of technical promotion) = Σnumber of fallowed households benefited by new forestry business entities × per capita cost of maintaining stability + Σarea of fallowed households benefited by new forestry business entities× average cost of forestry technology promotion

**Notes:** (1) According to the study by the World Bank, the coefficient of increase in forest employment is 2.2–4.2. Given that, after the development of forest economy and special economic forest on fallowed land, the ability of fallowed land to absorb labor force employment is enhanced, the median value of 3.2 is taken [34,35]; (2) Referring to the forest tourism industry driving coefficient of 3.58–5.97 in China from 2000 to 2011, the industry driving coefficient has been showing an increasing trend. Therefore, the highest value of 5.97 is taken for the past 5 years [36,37]; (3) Based on the long-term investment multiplier model, the long-term marginal consumption propensity of Chinese residents is 0.25, and the long-term investment multiplier is 1.33. Based on the short-term investment multiplier model, the short-term investment multiplier of Chinese residents is about 1.4. The long-term investment multiplier of 1.33 is chosen from the long-term investment consideration of the CFFG project [38]; (4) 0.13 of per capita medical cost. According to the survey, among more than 20 secrets of longevity, four to six of which are related to dietary health, the role of dietary nutrition on health is second only to genetics, and the health contribution rate is 13%, which is much larger than the role of medical factors [39,40]; (5) 0.3 of per capita medical costs, referring to the data on the relationship between the implementation of forest recreation and medical costs in Germany. According to relevant reports, Germany’s total national medical cost payment was reduced by 30% after the implementation of forest recreation programs [41]; (6) income multiplier for rural residents, according to the research results of “The multiplier effect of increasing income on GDP from the propensity to consume” [42]. The income multipliers for rural residents and urban residents are about 5 and 3, respectively, in China;(7) other average costs refer to China’s Statistical Yearbook, 2019 [43], China’s Rural Statistical Yearbook, 2019 [44], etc. All the calculations are based on the county level.

**Table 4:** National ecological benefits of the CFFG project in 2019.

Categories	Components	Value Volume (Million Yuan·y^−1^)	Percentage (%)
Supporting services	Soil conservation	129,851	9.16
	Accumulation of nutrients by forests	18,617	1.31
Regulating services	Water conservation	463,022	32.68
	Oxygen release and carbon sequestration	223,017	15.74
	Atmospheric purification	310,175	21.89
	Forest protection	65,435	4.62
Provisioning services	Biodiversity conservation	206,747	14.59
Total		1,416,864	100

**Table 5:** Economic value of the CFFG project in 2019.

Projects	Sub-Items	Value Volume (Million Yuan·y^−1^)	Percentage (%)
Primary Industry	Subtotal	148,305	58.05
	Value of economic forest products	84,223	
	Value of timber and bamboo harvesting	18,707	
	Value of forest plantation	36,630	
	Value of forest farming	8745	
Secondary Industry	Subtotal	65,453	25.62
	Value of timber processing	34,992	
	Value of forest chemical product manufacturing	6374	
	Woody oil seeds, fruits and vegetables, tea beverages, etc. processing and manufacturing value	20,363	
	Processing value of forest medicinal herbs	3724	
Tertiary Industry	Subtotal	41,728	16.33
	Forestry production service value	11,158	
	Forestry professional and technical service value	2986	
	Eco-tourism and forest recreation service value	27,584	
Total		255,486	100

**Table 6:** Social value of the CFFG project in 2019.

Projects	Sub-Items	Value Volume (Million Yuan·y^−1^)	Percentage (%)
Development of social undertakings	Subtotal	447,420	61.06
	Value of employment absorbed by farming retreat	142,868	
	Value of labor transfer from farming	192,060	
	Training value of farming fallow	886	
	Value of ecological education bases	12,236	
	Value of ecological cultural works	415	
	Ecological tourism value	98,955	
Optimization of social structure	Subtotal	247,959	33.84
	Village greening and beautification contribution value	99,079	
	Contribution value of fallow investment	28,227	
	Value of consumption of special economic forest products	15,397	
	Value of income from conversion of farmlands to forest	105,256	
Improving social services	Subtotal	6226	0.85
	Value of covering the poor population	5635	
	income from forest rangers	591	
Promoting the development of social organizations	Subtotal	31,091	4.24
	Scale value of new type of forestry management entities	29,633	
	Value of households benefited by fallowed new forestry business entities	1458	
Total		732,696	100

## Data Availability

Not applicable.
